# Fine-scale temporal and spatial variation of taxon and clonal structure in the *Daphnia longispina *hybrid complex in heterogeneous environments

**DOI:** 10.1186/1471-2148-12-12

**Published:** 2012-01-27

**Authors:** Mingbo Yin, Adam Petrusek, Jaromir Seda, Justyna Wolinska

**Affiliations:** 1 Ludwig-Maximilians-Universität, Department Biologie II, Evolutionsökologie, Großhaderner Str. 2, D-82152 Planegg-Martinsried, Germany; 2 Current address: Institute of Biodiversity Science, Fudan University, Shanghai 200433, China; 3 Charles University in Prague, Faculty of Science, Department of Ecology, Viničná 7, CZ-12844 Prague 2, Czech Republic; 4 Biological Centre AS CR, Institute of Hydrobiology, Na Sádkách 7, CZ-37005 České Budějovice, Czech Republic

## Abstract

**Background:**

Cyclical parthenogenetic water fleas of the genus *Daphnia *have become a prominent model organism in ecology and evolution. In the past, analyses of their population structure have been limited by the prevailing use of allozyme markers, which in general do not allow for the distinction of individual clones. In this study, we used 10 microsatellite markers to track changes in the taxonomic and clonal composition of *Daphnia *populations, and traced the abundance of the most common clones in two European reservoirs. One of the localities was inhabited by a single species of the *Daphnia longispina *complex (*D. galeata*), the other by two parental species (*D. galeata *and *D. longispina*) and their interspecific hybrids. The study took place during the transition from summer stratification to autumn mixing, representing a period of major environmental change within lake habitats.

**Results:**

In both reservoirs, we observed temporal (generation-to-generation) and spatial (along the heterogeneous reservoir environment) changes in *Daphnia *community structure. In the single-species reservoir, the clonal diversity of *D. galeata *increased with time, as a few dominant clones were replaced by a higher number of less common clones. A loss in selective advantage for the dominant clones may have been due to gradual changes in the environment, or due to selection acting in a negative frequency-dependent manner. In the multispecies reservoir, there were no apparent temporal trends in clonal diversity but we observed significantly lower clonal diversity in the interspecific hybrids than in the coexisting parental species, supporting the existence of reproductive barriers between the parental genomes.

**Conclusions:**

Our study, tracing clonal lineages of *Daphnia *in time and space by the fine-resolution markers, contributes to the understanding of how clonal reproduction impacts community structure in cyclically parthenogenetic organisms.

## Background

Cyclically parthenogenetic organisms, which reproduce both sexually and asexually, are common in nature, in both the animal and plant kingdoms [1,2]. This mode of reproduction, especially its asexual (clonal) component, has attracted considerable interests among population geneticists and evolutionary biologists. Asexual phase of the cyclically parthenogenetic reproduction cycle may have profound impact on population structures. For example, in the long-term, clonal reproduction may reduce the number of genetically distinct individuals within a population and hence decrease the effective population size (e.g. [3,4]). It may also lead to a spatial genetic autocorrelation, which could be falsely attributed to limited propagule dispersal or kin-structured colonization (e.g. [5]). Finally, as clones differ in fitness under varying environmental conditions [6], changes in clone frequencies are expected across the growing season [7]. Consequently, clonal selection can result in the strong reduction of clonal diversity [4,8].

In freshwater habitats, cyclical parthenogenesis is common among many groups of zooplankton [1]. Cladocerans, which reproduce parthenogenetically during favourable conditions and switch to sexual reproduction when conditions deteriorate (e.g. [9]), are particularly important in these environments, being the main component of aquatic food-webs [10,11]. The cladoceran genus *Daphnia *is commonly used as a model system for cyclical parthenogenesis in ecological and evolutionary research. In some *Daphnia *species complexes, interspecific hybrids may be produced during the sexual part of their reproductive cycle [12,13]. Hybridization has been documented within several species complexes of *Daphnia *from Eurasia, North America and Australia [12,13], but most research has concentrated on the *D. longispina *complex, inhabiting permanent lakes of the northern temperature zone [13]. In Europe, this complex includes, together with some rarer taxa, the widespread and ecologically important species *D. cucullata*, *D. galeata *and *D. longispina *[14]. These species often form interspecific hybrids which sometimes reach high abundances [13,15-18]. Once *Daphnia *hybrids are produced by sexual recombination, they can be maintained by clonal propagation for many generations [13], as in other cyclical parthenogens (e.g. [2,19]). In the *D. longispina *complex, although parental species also reproduce clonally for most of the year, there is evidence that they invest more into sexual reproduction than their F1 hybrids [16,20].

In previous studies of the *D. longispina *complex, the relative frequencies of different taxa were compared across time (e.g. [20-23]) and space (e.g. [15,17,24]). However, changes in clonal structure have been largely unexplored due to methodological limitations. So far, the most common method for identification of clones in the *D. longispina *complex has been allozyme electrophoresis (e.g. [23,25-28]), although RAPD markers were also used occasionally (e.g. [27]). However, allozyme studies are limited by the few polymorphic loci they provide; in most cases, it is likely that the multilocus genotypes defined by allozymes represented clonal groups [29]. This substantially limits the power to trace the frequencies of single clones and to study clonal structure in general. RAPDs, although more variable, have often poor reproducibility [30] and, being dominant markers which cannot separate homozygotes from heterozygotes [31], have limited use in the analyses of population structure. Recently, microsatellite markers have been developed for the *D. longispina *complex [32]. However, the subsequent studies employing these markers have focused so far on either a description of population state at a single time point [29,33] or on exploring temporal changes at the taxon level only [18,21,34]. In other systems, microsatellites have already been proven to be very powerful in tracing clonal lineages; for example, in the cyclically parthenogenetic aphid [35] or in bacterial populations [36].

In the present study, we used 10 microsatellite loci to explore temporal and spatial dynamics in the taxonomic and clonal structure of the *D. longispina *hybrid complex, in two reservoirs in the Czech Republic. The canyon-shaped morphology of these reservoirs creates longitudinal environmental gradients, which results in a spatial variation in the composition of zooplankton communities including *Daphnia *[17,24]. One of the studied reservoirs (Římov) was recently dominated by a single parental species (*D. galeata*), whereas three parental species (*D. galeata*, *D. longispina*, and *D. cucullata*) as well as their interspecific hybrids coexisted in the second reservoir (Vír) [17,24]. We screened *Daphnia *communities in these reservoirs at the end of the growing season, when temperate lakes undergo a major change - a transition from summer stratification to autumn mixing and winter conditions [10]. The goals of the study were to explore dynamics in taxonomic and clonal structure, across both time (generation-to-generation) and space (between sampling stations along the reservoir's longitudinal gradient), during a period of seasonal environmental change. We also tested one particular hypothesis that the clonal diversity is lower in hybrids than in parental species, due to some pre- and postzygotic barriers between parental genomes [16], resulting in a lower number of newly produced hybrids in comparison to parental clones.

## Methods

### Study site and field collections

*Daphnia *samples were collected from two man-made reservoirs in the Czech Republic: Římov (48°50'N, 14°30'E; constructed in 1978) and Vír (49°34'N, 16°19'E; constructed in 1959). Both reservoirs have canyon-shaped morphology, being elongated and meandering in deep valleys (for their outlines and further morphometric details see [17]). We analysed samples collected from three different stations along each reservoir's longitudinal axis; samples were taken by hauling a plankton net (mesh size 170 μm) through the entire water column, and preserved in 96% ethanol. The first sampling station was always located at the dam and the distance between sampling stations was about 4 km in Římov and 2 km in Vír. The three sampling stations are hereafter referred to as *dam*, *middle *and *upper*. To reveal fine-scale temporal variation in clonal composition, we aimed to repeat the sample collection when one generation time of the studied species had elapsed. *Daphnia *growth is strongly temperature-dependent (e.g. [37]), and although other factors, such as food availability (e.g. [38]), can also influence its growth rate, temperature changes play a major role in the studied period of the year. Therefore, we adjusted the sampling schedule to one *Daphnia *generation by calculating maturation time based on the surface water temperature [39] and experimental data [37]. Each station was sampled five times between September 14 (end of the summer stratification period) and December 9, 2009 (onset of winter) but, as cooling continued throughout autumn, the sampling intervals became longer (see Table 1 in [40]).

### Sample processing and microsatellite genotyping

Using the stereomicroscope, ca. 94 adult females from the *D. longispina *complex were randomly chosen from each time point and station for genetic analyses. The *upper *station in Římov had not been considered at the last time point (i.e. *t+4*) because of a very low *Daphnia *density. Additionally, we examined other 100-300 adult females per sample for the presence of ephippia, which indicate a switch from parthenogenetic to sexual reproduction. All individuals were genotyped at 10 previously published microsatellite markers [32] in a multiplex polymerase chain reaction (Dgm109, Dp196, Dp281, Dp512, SwiD1, SwiD2, SwiD10, SwiD12, SwiD14, SwiD15), by using Multiplex PCR Kit (Qiagen). Detailed protocol has already been described elsewhere [18]. Genotypes were checked by GeneMapper version 3.7 (Applied Biosystems). Before data sets from different plates were merged, the consistency of alleles was checked against loci-specific patterns of a reference clone used in each run, which allowed us to appropriately score alleles with small differences in fragment lengths. In addition, there was no evidence that scoring errors resulted from stuttering, large allele dropout or presence of null alleles, as indicated by tests (10^4 ^permutations) in MICRO-CHECKER 2.2.3 [41].

### Data analyses

#### Taxon assignment

The similarity of multilocus genotypes (MLGs) characterised by alleles at 10 microsatellite loci was first displayed by the factorial correspondence analysis (FCA), performed in GENETIX 4.05 [42], in which each different MLG was represented by one individual. As reference parental species, we used 40 well-defined genotypes of *D. cucullata*, *D. galeata *and *D. longispina*, originating from 23 locations in Europe and one location in North America, which were also classified using two allozyme loci, recognised to be diagnostic for species identification [33,43]. For detailed information about those genotypes see [18]. NewHybrids 1.1 [44], run for 10^6 ^iterations after a burn-in of 10^6 ^length, was used to assign individuals from field samples into taxonomic units based on their MLGs. Taxon membership was identified by applying a threshold of 95% posterior probability to assign individuals to one of six predefined categories: two parental species, two backcross groups, or F1 and F2 hybrids. Furthermore, we used logistic regression to test if the distribution of unidentified individuals (below the threshold of 95%) was different among samples; assignment (i.e. identified vs. unidentified) was treated as a dependent variable and the samples (categorical data) as a covariate. The calculations of clonal diversity within taxa (see below) were based on the taxon assignment from NewHybrids.

#### Clonal assignment

First, we calculated the *P*_sex _index [45] which determines the likelihood of a clone encountered more than once as being a result of sexual recombination, instead of clonal propagation (GENCLONE 2.0, [46]). In case of crossing between common clones (or selfing within a clone), the likelihood to encounter identical MLGs would be substantially higher than *P*_sex _suggests (under random mating); however, this should not change the interpretation of our data, since we assume such genetically similar sibling (if present in our dataset) might also have similar ecological characteristics. We also confirmed the resolution power of the used microsatellite markers by genotyping forty *D. galeata *individuals from Římov at five additional loci, with only little increase in observed clonal richness (see [40]).

#### Comparison of clonal diversity between hybrid and parental taxa

Clonal diversity, MLG/N (number of MLG divided by sample size), was calculated for each population sample (defined as a group of individuals belonging to the same taxon, and found at a given time and station) with a minimum sample size of 10 individuals (GENALEX 6 [47]). All individuals with missing data at any of the loci were excluded (except the *D. longispina *individuals from Vír with missing data at the locus SwiD2; see Results). Then, the clonal diversity was compared between each of the two parental species (*D. galeata *and *D. longispina*) and their F1 hybrids co-occurring at the same time and sampling station (using the Wilcoxon test paired across sampling dates and stations).

#### Temporal and spatial variation in taxon composition

Using data from Vír, we applied a multinomial generalised linear model (GLM) in R [48], to test the effects of time (i.e. five time points), space (i.e. three stations) and their interaction term (time × space) on taxon composition; the response was a matrix with five columns (i.e. five classes: *D. galeata*, *D. longispina*, F1 hybrids, backcross to *D. longispina *and unidentified). The command "anova.multinom" was used to perform analyses of deviance. In addition, we tested for the effect of time, space and their interaction on a frequency of a certain taxon (against the frequency of all other taxa in a given sample), by applying the binomial GLM in R. For the model selection, we used a backward elimination procedure, removing the least significant factors (*P *> 0.15) from a parameter-rich model. These analyses were performed for the most common taxa encountered in Vír (*D. galeata*, *D. longispina *and F1 hybrids). Sequential Bonferroni corrections were applied when interpreting the results.

#### Temporal and spatial variation in clonal composition

For both reservoirs, we investigated temporal and spatial changes in clonal composition within taxa. For these analyses, the clones which did not exceed a frequency of 10% in at least one sample of N ≥ 10, were pooled and labelled as "rare". First, similar multinomial GLMs as described above were applied to test the effects of time, space and their interaction (as fixed factors) on clonal composition within taxa and reservoir (i.e. on the response matrix containing the common clones and the "rare" category). Then, the binomial GLM was applied to test whether the proportion of each particular "common" clone is stable (in time or space), by comparing the frequency of a certain clone against the frequency of all other clones in a given sample (additionally included here were three samples of N = 8 for F1 hybrids and one sample of N = 7 for *D. longispina*). Sequential Bonferroni corrections were applied accordingly.

#### Temporal and spatial variation in clonal diversity

We tested the effects of time, space and their interaction term on clonal diversity (for samples with N ≥ 10), by applying a GLM (family = Gaussian). In order to partition the genetic variance into temporal, spatial and within-sample components, we applied an analysis of molecular variance (AMOVA; N ≥ 10; performed in Arlequin 3.0 [49], using 10^4 ^permutations).

## Results

### Sexual reproduction

The studied *Daphnia *populations did not switch to sexual reproduction during the study period. Among the 3136 females examined from Vír, only nine (0.3%) females with ephippia were detected. In Římov, not a single sexual female was detected among 2861 examined individuals. Moreover, we only observed very few *Daphnia *males (even lower proportion than those of ephippial females).

### Taxon assignment

**Figure 1:**
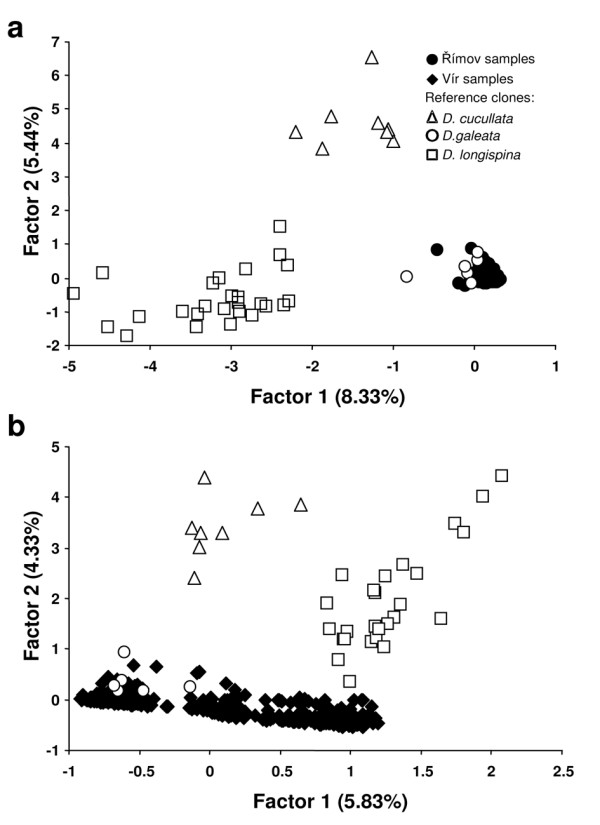
**Factorial correspondence analysis showing genetic variation among analysed individuals, based on allelic variation at 10 microsatellite loci**. Each data point represents a multilocus genotype from the 40 reference clones and the reservoirs *Daphnia *from a) Římov and b) Vír.

Results of the Factorial Correspondence Analysis confirmed that the two reservoirs substantially differed in *Daphnia *taxon composition. All 1254 genotyped *Daphnia *from Římov clustered around the reference clones of *D. galeata *(Figure 1a). In contrast, the distribution of 1386 genotyped *Daphnia *from Vír in the FCA plot indicated a presence of individuals of more diverse parentage. Some of these also clustered around the reference clones of *D. galeata*, the others were positioned in between the clusters representing two parental species: *D. galeata *and *D. longispina *(Figure 1b). The Bayesian analyses implemented with NewHybrids also assigned MLGs from Římov to one taxon, *D. galeata *(with only one *Daphnia *remaining unidentified with a 95% posterior probability threshold). The MLGs from Vír were assigned to five classes: *D. galeata *(52.6% of all individuals), *D. longispina *(16.5%), backcross to *D. longispina *(3.1%), F1 (20.8%) and F2 hybrids (a single individual; 0.1%). However, 95 individuals (i.e. 6.9%) remained unidentified (Additional file 1). Even when the threshold of 80% posterior probability was applied, 51 *Daphnia *still remained unidentified; the remaining *Daphnia *were identified mainly as F1 hybrids (25 individuals) or as *D. longispina *(12 individuals). The unidentified individuals (threshold of 95%) were equally distributed across the samples (*P *= 0.16), and were thus excluded from further analyses that required identification of the individual to taxon level.

### Clonal assignment

In Římov, among the 1220 individuals with complete MLG profiles, 392 unique MLGs were detected. In Vír, among 1329 individuals with complete MLG or data missing solely at the SwiD2 locus (many *D. longispina *individuals could not be amplified at this locus but the amplification worked well at the remaining nine loci), 587 unique MLGs were found. There was not a single MLG shared between the two reservoirs. As the *P*_sex _value was lower than 10^-5 ^across all six performed tests (i.e. two reservoirs × three sampling stations), we considered individuals sharing the same multilocus genotype at all evaluated loci as belonging to the same clone.

### Comparison of clonal diversity between hybrid and parental taxa

In Vír, where two parental taxa and their recombinant genotypes were present, F1 hybrids had significantly lower clonal diversity (MLG/N) than *D. galeata *(0.46 ± 0.15 SD vs. 0.74 ± 0.10; N = 12, *Z *= 3.06, *P *= 0.002) or *D. longispina *(0.51 ± 0.13 vs. 0.74 ± 0.21; N = 7, *Z *= 2.03, *P *= 0.042; Additional file 1), which co-occurred with the hybrids at the same time and at the same sampling stations. After excluding the SwiD2 locus, the pattern remained the same (data not shown). Although the number of individuals taken for calculations of clonal diversity was, on average, smaller for F1 hybrids than for *D. galeata *(*P *< 0.001; paired t-test), it did not differ between F1 hybrid and *D. longispina *(*P *= 0.46); thus, the variable sample sizes should not be a cause for the observed lower diversity in F1 hybrids.

### Temporal and spatial variation in taxon composition

**Figure 2:**
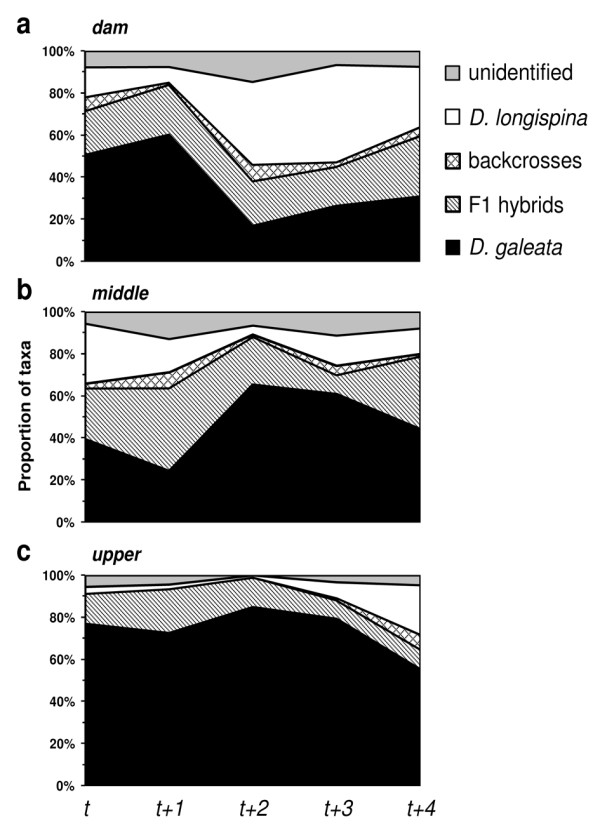
**Changes in the taxon composition of *Daphnia *communities in the Vír reservoir at each of the three sampling stations: a) *dam*, b) *middle *and c) *upper***. Taxon classification is based on the NewHybrids assignment; a single F2 hybrid is pooled with unidentified individuals (below a threshold of 95% posterior probability).

In Vír, the taxon composition of the *Daphnia *community changed significantly across time (i.e. five subsequent *Daphnia *generations) and space (i.e. three stations); whereas the interaction effect was only marginally significant (multinomial GLM, Additional file 2). Considering changes in the frequencies of single taxa (vs. other taxa, binomial GLMs), the frequencies of *D. galeata *and *D. longispina *changed across time and space, whereas the frequencies of the F1 hybrids changed across time only, and there was a significant time × space interaction (Figure 2, Table 1). Interestingly, the observed temporal changes in *D. galeata *were opposite between *dam *and *middle *stations. At the *dam *station, the proportion of *D. galeata *decreased from 60% to 17% between *t+1 *and *t+2 *(Figure 2a). In contrast, the proportion of this species increased from 24% to 60% during the same time period at the *middle *station (Figure 2b).

**Table 1:** Changes in relative taxon frequency across time and space in Vír.

Taxon	Time		Space		Time × Space	
	*Z*	*P*	*Z*	*P*	*Z*	*P*
*D. galeata*	-2.42	**0.015**	3.12	**0.002**	1.73	0.084
F1 hybrids	2.18	0.029		ns	-3.8	**< 0.001**
*D. longispina*	3.71	**< 0.001**	-7.76	**< 0.001**		ns

The fitted binomial GLM model is shown (terms removed from the model are labelled as 'ns'). The *P*-values that remained significant after sequential Bonferroni correction are marked in bold (α = 0.05/3).

### Temporal and spatial variation in clonal composition

**Figure 3:**
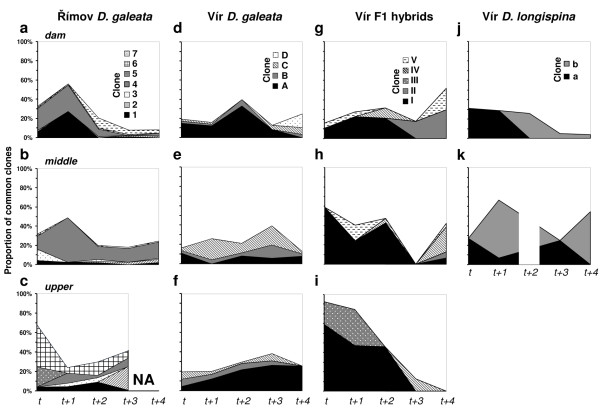
**Changes in clonal composition of *Daphnia *taxa in Římov and Vír reservoirs at each of the three sampling stations (*dam*, *middle *and *upper*)**. Distribution of the most common clones (i.e. frequency > 10% in at least one sample) is shown. Remaining clones were pooled and classified as "rare" (white area, up to 100%). NA indicates dates when *Daphnia *were no longer present at the sampling site. A blank square across the graph indicates a date when there were too few individuals of the species available to calculate clone frequencies (n = 4).

In order to trace the clonal lineages, seven *D. galeata *clones from Římov, and four *D. galeata*, five F1 hybrid and two *D. longispina *clones from Vír were selected as a set of "common" clones (with frequency exceeding 10% in at least one sample), whereas the other clones were pooled together into the "rare" group (Figure 3). *D. longispina *from Vír were only analysed at the *dam *and *middle *stations because of their low frequency at the *upper *station (see Figure 2). For *D. galeata *from Římov and the three taxa from Vír, significant changes in clonal composition were detected across time (i.e. five subsequent *Daphnia *generations; *P *< 0.01), space (i.e. three stations; *P *< 0.01) and their interaction (i.e. time × space; *P *< 0.05; Additional file 3).

In the binomial GLM model, six *D. galeata *clones from Římov, as well as three *D. galeata*, three F1 hybrid and two *D. longispina *clones from Vír were analysed (the remaining "common" clones were only detected at one or two sampling dates/stations, resulting in too many zero values to be included in the dataset). In Římov, four out of the six tested common *D. galeata *clones showed significant changes across time, five clones across space, and three clones showed a significant time × space interaction (Table 2). For example, the proportion of clone 4 at the *dam *station remained constant at ca. 25% for the first two time points (i.e. at *t *and *t+1*) and then decreased to below the detection limit at *t+3 *(i.e. 42 days later; Figure 3a). In the *middle *station, the proportion of clone 4 increased from 14% at *t *to 46% at *t+1 *(i.e. within 15 days), then decreased and fluctuated afterwards (around 15%; Figure 3b). In the *upper *station, the proportion of clone 4 was much lower and fluctuated with time (from below detection to 10%; Figure 3c).

**Table 2:** Changes in relative frequencies of common clones across time and space, in Římov and Vír (calculated per *Daphnia *taxon).

Taxon (reservoir)	clone ID	Time		Station		Time × Space	
		*Z*	*P*	*Z*	*P*	*Z*	*P*
*D. galeata *(Římov)	1	-3.38	**< 0.001**	-2.87	**0.004**	2.10	0.035
	2	-2.83	**0.004**	-2.66	**0.007**	3.73	**< 0.001**
	3	-2.83	**0.005**		ns	1.88	0.059
	4	-5.52	**< 0.001**	-5.48	**< 0.001**	4.73	**< 0.001**
	6		ns	8.52	**< 0.001**	-5.57	**< 0.001**
	7		ns	-3.46	**< 0.001**	1.99	0.046
*D. galeata *(Vír)	A	-3.39	**< 0.001**	-2.93	**0.003**	4.1	**< 0.001**
	B		ns	1.28	0.198		ns
	C	0.51	0.613		ns		ns
F1 hybrids (Vír)	I	-5.21	**< 0.001**	4.04	**< 0.001**		ns
	IV		ns	-2.07	0.038	3.07	**0.002**
	V	2.51	**0.012**		ns	-2.13	0.030
*D. longispina *(Vír)	a	-2.72	**0.006**	-1.83	0.067	2.32	**0.020**
	b	-1.93	0.052		ns	3.12	**0.001**

The fitted binomial GLM model is shown (terms removed from the model are labelled as 'ns'). The *P*-values that remained significant after sequential Bonferroni correction are marked in bold.

In the *D. galeata *population from Vír, one of the three common clones showed significant changes across time and space and a significant interaction effect (Table 2). Among the F1 hybrids, one of the three tested clones showed significant changes across time and space; specifically, clone I was most abundant at time *t *in both the *middle *(59%, Figure 3h) and *upper *stations (69%, Figure 3i), but was rare at the *dam *station (10%, Figure 3g). The frequency of this clone consistently decreased towards the end of the growing season at all three stations. A second clone differed across time only, whereas a significant time × space interaction was detected for a third clone (Table 2). Among the *D. longispina *clones, one of the two tested clones showed significant changes across time, and both clones showed a significant interaction effect (Table 2).

### Temporal and spatial variation in clonal diversity

**Figure 4:**
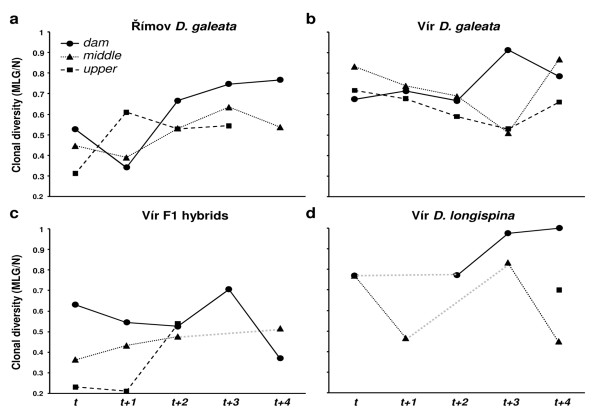
**Changes in clonal diversity (number of distinct multilocus genotypes/sample size) of a) *D. galeata *in the Římov reservoir, and b) *D. galeata*, c) F1 hybrid and d) *D. longispina *populations in the Vír reservoir, at each of the three sampling stations (*dam*, *middle *and *upper*)**. Only samples with n ≥ 10 are shown, grey dotted lines connect results from non-adjacent sampling dates.

In the Římov reservoir, the clonal diversity of the *D. galeata *population increased significantly with time (Figure 4a, Table 3). In the Vír reservoir, however, there was no apparent trend in any of the three tested taxa (Figure 4b-d). Even after pooling all the taxa into an "all-*Daphnia*" dataset, no temporal trend in clonal diversity was detected (data not shown). In the F1 hybrids, the clonal diversity differed significantly among stations; it was low at the *upper *station, intermediate at the *middle *station and high at the *dam *station (Figure 4c).

**Table 3:** Changes in clonal diversity across time and space, in Římov and Vír (calculated per *Daphnia *taxon).

Taxon (reservoir)	Time		Space		Time × Space	
	*F*	*P*	*F*	*P*	*F*	*P*
*D. galeata *(Římov)	7.78	**0.018**	1.76	0.21	0.07	0.79
*D. galeata *(Vír)	0.03	0.86	2.90	0.11	1.93	0.19
F1 hybrids (Vír)	0.67	0.43	13.49	**0.006**	9.05	**0.016**
*D. longispina *(Vír)	2.08	0.22	1.91	0.24	0.01	0.94

All significant values are marked in bold.

The AMOVA indicated that most of the genetic variance was explained by within-sample variation (from 92% to 99%, Table 4). The remaining variation was more or less equally distributed between temporal and spatial components. These components were in all but one case significant; the exception being a spatial component for *D. galeata *in Vír. This corresponded well to the results of the binomial GLM tests (see above), in which clonal frequency differed in space for one of the three common *D. galeata *clones only (Table 2).

**Table 4:** Hierarchical analysis of molecular variance (AMOVA) among sampling stations and within time points (calculated per *Daphnia *taxon).

Taxon(reservoir)	Source of variation	DF	Explained variation	*P *
*D. galeata *(Římov)	Across space	2	0.95%	**0.038**
	Across time (within space)	11	4.13%	**< 0.001**
	Within sample	2494	94.9%	**< 0.001**
*D. galeata *(Vír)	Across space	2	0.00%	0.297
	Across time (within space)	12	0.93%	**< 0.001**
	Within sample	1425	99.1%	**< 0.001**
F1 hybrids (Vír)	Across space	2	2.64%	**< 0.001**
	Across time (within space)	9	1.45%	**< 0.010**
	Within sample	508	95.9%	**< 0.001**
*D. longispina *(Vír)	Across space	2	5.53%	**< 0.001**
	Across time (within space)	6	2.92%	**< 0.001**
	Within sample	395	91.6%	**< 0.001**

## Discussion

Using a set of recently developed high-resolution microsatellite markers [32], we observed significant changes in *Daphnia *taxon and/or clonal composition and diversity on a very fine temporal and spatial scale, for two different *Daphnia *communities. Specifically, the sampling intervals were adjusted to a single *Daphnia *generation (i.e. 14-30 days, depending on temperature) and the spatial distribution of taxa and clones were studied within individual water bodies.

In contrast to the Vír reservoir, where two parental species (*D. galeata *and *D. longispina*) and three hybrid classes (backcrosses, F1 and F2) were detected, only one taxon (*D. galeata*) was found in Římov. These results correspond to previous allozyme-based studies of *Daphnia *communities in the same reservoirs [17,24]. Differences in the *Daphnia *taxon composition between environmentally similar habitats are not extraordinary: both the coexistence of several taxa from the *D. longispina *complex and the dominance of one taxon have been reported across many European lakes (e.g. [15,18,23]). In Vír, we observed temporal and spatial taxonomic shifts during the seasonal transition. The observed shifts may be explained by the impact of alternations in environmental conditions on the relative fitness of taxa. Previous experimental studies on the *D. longispina *complex have shown that relative taxon fitness varies with food quality (e.g. [50]), predation (e.g. [51]) and parasite pressure [52]. Indeed, differences in local food conditions and predation regimes are considered as drivers of spatial differentiation in taxon composition within reservoirs [17,24]. Moreover, we have recently observed that parasite pressure also varies across time and space in these reservoirs [53].

In addition to affecting the distribution of *Daphnia *species and hybrids, environmental heterogeneity may also affect competition at the clonal level. Although some clones were shared among the sampling dates and stations, their relative frequencies often substantially differed (similarly as reported in a recent study [54] which used allozyme markers). This is in agreement with the experimental studies on the *D. longispina *complex which revealed that the relative performance of clones varies across environmental conditions, even within a single taxon (e.g. [50-52]). Moreover, for one taxon (F1 hybrids in Vír), the clonal diversity also differed across the stations in a consistent manner; being high at the *dam*, intermediate at the *middle *and low at the *upper *station. Various scenarios may explain such patterns. First, some clones detected at the *dam *station could have been passively dispersed from the upper parts of the reservoir; i.e., the dam region might serve as a sink that accumulates more genotypes. Second, there is an environmental gradient within the reservoir even at the end of the season and spatial differences in local condition could promote different clonal diversity in some taxa.

In general, populations of cyclical parthenogens are expected to have high clonal diversity at the start of the growing season due to the hatching of new genotypes. Conversely, during periods of asexual reproduction, the extinction of clones due to selection and random events should lead to an erosion of clonal diversity [8,55]. For example, allozyme studies have detected a decrease in clonal diversity in *D*. *magna *and *D*. *pulex *populations inhabiting temporary ponds during the course of a single growing season [56,57] and similar patterns were reported for other cyclical parthenogens such as rotifers (e.g. [58]) and aphids [59]. However, for the transition period between summer stratification and autumn mixing, which happens at the end of the growing season, our data showed an increase in clonal diversity with time for the *D. galeata *from Římov, and clonal diversity remained roughly constant in *D. galeata*, *D. longispina *and hybrids from Vír. This suggests that changes in selection pressures during this period did not result in further clonal erosion.

The observed decrease in the frequency of some common clones (which might result in an increase in clonal diversity) could potentially result from an investment into sexual rather than parthenogenetic reproduction. However, this was not the case in the studied reservoirs; as the proportion of ephippial females and males was negligible. Rather, it seems that in Římov, a few *D. galeata *clones that were dominant at the beginning of autumn lost their relative competitive advantage and were later replaced by a higher number of otherwise less common clones. The clones that were favoured during stratified summer conditions may have relatively lower fitness in low food and low temperature, while other clones may be better adapted to these harsh environments. Such clones apparently are present in reservoir environments. For example, a recent study observed a genetically differentiated hypolimnetic population of *D. galeata *in Římov [26] with different life-history traits than their epilimnetic counterparts [60]. Clones originating from the deep hypolimnion, where the water is colder and less nutrient-rich, may have an advantage at the end of the growing season. The decrease in the abundance of common clones could also be caused by selection pressure acting in a negative frequency-dependent manner. For example, there is some evidence from a field survey of the *D. longispina *complex communities that common clones are attacked by coevolving parasites, which consequently reduce their frequencies [28]. As the prevalence of *Daphnia *parasites is high in both reservoirs, especially in autumn [40,53], and these parasites do not infect all clones equally [40], it is possible that at least some common clones are handicapped by parasite-driven, time-lagged negative frequency-dependent selection (see [61]).

Finally, the significant difference in clonal diversity between co-occurring taxa in Vír supports our hypothesis that F1 hybrids have lower clonal diversity than parental species. This is consistent with the results from a recent field survey of communities of the *D. longispina *complex across several small lakes in Germany, where the clonal diversity of F1 hybrids was lower than that of parental species [18]. In that previous study, however, clonal diversity was compared between the taxa that originated from different water bodies, so it could not be excluded that the observed patterns were partially caused by habitat differences. In another field survey of a *D. longispina *community across a single lake, significantly fewer hybrid genotypes were detected in sexually-produced diapausing eggs (ephippia) than would be expected if mating were random; furthermore, hybrid diapausing embryos were shown to have lower hatching success than parental ones [16]. Thus, the present study contributes another evidence for the existence of some reproductive incompatibilities between the parental genomes of hybridizing species in the *D. longispina *complex.

## Conclusions

We have detected, on a very small sampling scale, significant temporal and spatial changes in taxonomic and clonal composition in communities of the *D. longispina *hybrid complex. Analysis of 10 microsatellite loci allowed us to trace clonal lineages with unprecedented precision, in contrast to previous studies using very broad, allozyme-defined clonal groups (e.g. [23,25,43,44,62]). Our data show the replacement of dominant clones over a very short time period (within one or two generation times) and spatial genetic differentiation within single water bodies. On the other hand, we detected the presence of certain clones in substantial frequencies at sampling stations separated by several kilometres. Apparently, successful genotypes reach high densities and occupy vast areas within the reservoir despite the variation in selection pressure. Most likely, these common clones overwinter in the reservoir, which allows them to compete with other genotypes for extended periods of time and gain large proportions in the population (see also [4]). Altogether, our work highlights detailed changes in clonal structure within the *D. longispina *hybrid complex and contributes to understanding how clonal reproduction impacts community composition in cyclically parthenogenetic organisms.

## Authors' contributions

JW, AP and JS designed the study, and AP and JS conducted field sampling. MY carried out the molecular work. MY, JW and AP contributed to data analyses and preparation of the manuscript. All authors read and approved the final version.

## Supplementary Material

Additional file 1 **Clonal diversity of *Daphnia *populations, as calculated from 10 microsatellite loci**. A table lists the sampling information, sample size and clonal diversity for each sample. Click here for file

Additional file 2 **The effects of time, space and their interaction on the taxon composition in the Vír reservoir**. The table shows the results of the multinomial generalised linear model (GLM), analysed in the R package, testing the effects of time (i.e. five time points), space (i.e. three stations) and their interaction term (time × space) on taxon composition (i.e. five classes resulting from the NewHybrids analyses: *D. galeata*, *D. longispina*, F1 hybrids, backcross to *D. longispina *and unidentified). The command "anova.multinom" was used to perform analyses of deviance. Click here for file

Additional file 3 **The effects of time, space and their interaction on the clonal composition within taxa in Římov and Vír reservoirs**. The table shows the results of multinomial generalised linear models, analysed in the R package, testing the effects of time, space and their interaction (as fixed factors) on clonal composition within taxa in each reservoir (i.e. on the response matrix containing the common multilocus genotypes and the "rare" category). The command "anova.multinom" was used to perform analyses of deviance. Click here for file
