# Supplementary material for: Fine-scale temporal and spatial variation of taxon and clonal structure in the Daphnia longispina hybrid complex in heterogeneous environments
Source: BMC Evol Biol. 2012 Jan 27;12:12. doi: 10.1186/1471-2148-12-12 (PMC3305588; doi:10.1186/1471-2148-12-12)
Supplement: Additional file 1 — Clonal diversity of Daphnia populations, as calculated from 10 microsatellite loci. A table lists the sampling information, sample size and clonal diversity for each sample. [file 1471-2148-12-12-S1.DOC]

Additional file 1: **Clonal diversity of *Daphnia* populations, as calculated from 10 microsatellite loci.**

A table lists the sampling information, sample size and clonal diversity for each sample.

| Reservoir | Station | Sampling date | Taxon *^a^* | N *^b^* | N *^c^* | MLG | MLG/N*^c^* |
| --- | --- | --- | --- | --- | --- | --- | --- |
|
|  | *dam* | *t* |  | 93 | 91 | 48 | 0.53 |
|  |  | *t+1* |  | 91 | 91 | 31 | 0.34 |
|  |  | *t+2* | *D. galeata* | 88 | 87 | 58 | 0.67 |
|  |  | *t+3* |  | 88 | 87 | 65 | 0.75 |
|  |  | *t+4* |  | 78 | 60 | 46 | 0.77 |
|  | *middle* | *t* |  | 94 | 94 | 42 | 0.45 |
|  |  | *t+1* |  | 85 | 82 | 32 | 0.39 |
| Římov |  | *t+2* | *D. galeata* | 94 | 94 | 50 | 0.53 |
|  |  | *t+3* |  | 85 | 82 | 52 | 0.63 |
|  |  | *t+4* |  | 94 | 93 | 50 | 0.54 |
|  | *upper* | *t* |  | 94 | 93 | 29 | 0.31 |
|  |  | *t+1* | *D. galeata* | 89 | 87 | 53 | 0.61 |
|  |  | *t+2* |  | 88 | 87 | 46 | 0.53 |
|  |  | *t+3* |  | 93 | 92 | 50 | 0.54 |
| Vír | *dam* | *t* | *D. galeata* | 46 | 46 | 31 | 0.67 |
|  |  |  | F1 hybrids | 19 | 19 | 12 | 0.63 |
|  |  |  | *D. longispina* | 13 | 13 | 10 | 0.77 |
|  |  |  | unidentified | 7 | 6 | 4 | nc |
|  |  | *t+1* | *D. galeata* | 56 | 56 | 40 | 0.71 |
|  |  |  | F1 hybrids | 22 | 22 | 12 | 0.55 |
|  |  |  | *D. longispina* | 7 | 7 | 6 | nc |
|  |  |  | unidentified | 7 | 5 | 3 | nc |
|  |  | *t+2* | *D. galeata* | 15 | 15 | 10 | 0.67 |
|  |  |  | F1 hybrids | 19 | 19 | 10 | 0.53 |
|  |  |  | *D. longispina* | 35 | 35 | 27 | 0.77 |
|  |  |  | unidentified | 13 | 13 | 7 | 0.54 |
|  |  | *t+3* | *D. galeata* | 24 | 23 | 21 | 0.91 |
|  |  |  | F1 hybrids | 17 | 17 | 12 | 0.71 |
|  |  |  | *D. longispina* | 42 | 41 | 40 | 0.98 |
|  |  |  | unidentified | 5 | 5 | 5 | nc |
|  |  | *t+4* | *D. galeata* | 29 | 28 | 22 | 0.79 |
|  |  |  | F1 hybrids | 27 | 27 | 10 | 0.37 |
|  |  |  | *D. longispina* | 27 | 27 | 27 | 1.00 |
|  |  |  | unidentified | 7 | 7 | 3 | nc |
|  | *middle* | *t* | *D. galeata* | 36 | 36 | 30 | 0.83 |
|  |  |  | F1 hybrids | 22 | 22 | 8 | 0.36 |
|  |  |  | *D. longispina* | 26 | 26 | 20 | 0.77 |
|  |  |  | unidentified | 5 | 5 | 5 | nc |
|  |  | *t+1* | *D. galeata* | 23 | 23 | 17 | 0.74 |
|  |  |  | F1 hybrids | 37 | 37 | 16 | 0.43 |
|  |  |  | *D. longispina* | 15 | 15 | 7 | 0.47 |
|  |  |  | unidentified | 12 | 12 | 7 | 0.58 |
|  |  | *t+2* | *D. galeata* | 61 | 61 | 42 | 0.69 |
|  |  |  | F1 hybrids | 21 | 21 | 10 | 0.47 |
|  |  |  | *D. longispina* | 4 | 4 | 4 | nc |
|  |  |  | unidentified | 6 | 5 | 5 | nc |
|  |  | *t+3* | *D. galeata* | 55 | 51 | 26 | 0.51 |
|  |  |  | F1 hybrids | 8 | 8 | 7 | nc |
|  |  |  | *D. longispina* | 13 | 12 | 10 | 0.83 |
|  |  |  | unidentified | 10 | 6 | 5 | nc |
|  |  | *t+4* | *D. galeata* | 40 | 38 | 33 | 0.89 |
|  |  |  | F1 hybrids | 31 | 31 | 16 | 0.52 |
|  |  |  | *D. longispina* | 11 | 11 | 5 | 0.45 |
|  |  |  | unidentified | 7 | 6 | 5 | nc |
|  | *upper* | *t* | *D. galeata* | 70 | 67 | 48 | 0.72 |
|  |  |  | F1 hybrids | 13 | 13 | 3 | 0.23 |
|  |  |  | *D. longispina* | 3 | 3 | 2 | nc |
|  |  |  | unidentified | 5 | 1 | 1 | nc |
|  |  | *t+1* | *D. galeata* | 66 | 65 | 44 | 0.68 |
|  |  |  | F1 hybrids | 19 | 19 | 4 | 0.21 |
|  |  |  | *D. longispina* | 2 | 2 | 1 | nc |
|  |  |  | unidentified | 4 | 3 | 3 | nc |
|  |  | *t+2* | *D. galeata* | 79 | 78 | 46 | 0.59 |
|  |  |  | F1 hybrids | 13 | 13 | 7 | 0.54 |
|  |  |  | *D. longispina* | 1 | 1 | 1 | nc |
|  |  |  | unidentified | 0 |  |  |  |
|  |  | *t+3* | *D. galeata* | 73 | 68 | 36 | 0.53 |
|  |  |  | F1 hybrids | 8 | 8 | 8 | nc |
|  |  |  | *D. longispina* | 7 | 7 | 7 | nc |
|  |  |  | unidentified | 3 | 2 | 2 | nc |
|  |  | *t+4* | *D. galeata* | 47 | 47 | 31 | 0.66 |
|  |  |  | F1 hybrids | 8 | 8 | 6 | nc |
|  |  |  | *D. longispina* | 20 | 20 | 14 | 0.70 |
|  |  |  | unidentified | 4 | 4 | 4 | nc |

*^a^* the taxon membership was defined by the NewHybrids software based on the allelic variation at 10 microsatellite loci (only a few individuals were classified to the backcross taxa, and those are not included here); N *^b^*, total number of individuals; N *^c^*, number of individuals excluding missing data (in case of *D. longispina* individuals from the Vír reservoir, individuals with the missing data at the locus SwiD2 are included); MLG, number of unique multi-locus genotypes; nc, not calculated.
