# Supplementary material for: Fine-scale temporal and spatial variation of taxon and clonal structure in the Daphnia longispina hybrid complex in heterogeneous environments
Source: BMC Evol Biol. 2012 Jan 27;12:12. doi: 10.1186/1471-2148-12-12 (PMC3305588; doi:10.1186/1471-2148-12-12)
Supplement: Additional file 2 — The effects of time, space and their interaction on the taxon composition in the Vír reservoir. The table shows the results of the multinomial generalised linear model (GLM), analysed in the R package, testing the effects of time (i.e. five time points), space (i.e. three stations) and their interaction term (time × space) on taxon composition (i.e. five classes resulting from the NewHybrids analyses: D. galeata, D. longispina, F1 hybrids, backcross to D. longispina and unidentified). The command "anova.multinom" was used to perform analyses of deviance. [file 1471-2148-12-12-S2.DOC]

Additional file 2: **The effects of time, space and their interaction on the taxon composition in the Vír reservoir**.

The table shows the results of the multinomial generalised linear model (GLM), analysed in the R package, testing the effects of time (i.e. five time points), space (i.e. three stations) and their interaction term (time × space) on taxon composition (i.e. five classes resulting from the NewHybrids analyses: *D. galeata*, *D. longispina*, F1 hybrids, backcross to *D. longispina* and unidentified). The command “anova.multinom” was used to perform analyses of deviance.

| Model | Residual DF | Residual Deviance | DF | LR statistic | *P* |
| --- | --- | --- | --- | --- | --- |
| time and space | 24 | 2232.2 |  |  |  |
| time, space and their interaction | 22 | 2226.5 | 2 | 5.73 | 0.06 |
| space | 26 | 2246.3 |  |  |  |
| time and space | 24 | 2232.2 | 2 | 14.09 | **< 0.001** |
| time | 26 | 2354.9 |  |  |  |
| time and space | 24 | 2232.2 | 2 | 122.71 | **< 0.001** |
