# Supplementary material for: Fine-scale temporal and spatial variation of taxon and clonal structure in the Daphnia longispina hybrid complex in heterogeneous environments
Source: BMC Evol Biol. 2012 Jan 27;12:12. doi: 10.1186/1471-2148-12-12 (PMC3305588; doi:10.1186/1471-2148-12-12)
Supplement: Additional file 3 — The effects of time, space and their interaction on the clonal composition within taxa in Římov and Vír reservoirs. The table shows the results of multinomial generalised linear models, analysed in the R package, testing the effects of time, space and their interaction (as fixed factors) on clonal composition within taxa in each reservoir (i.e. on the response matrix containing the common multilocus genotypes and the "rare" category). The command "anova.multinom" was used to perform analyses of deviance. [file 1471-2148-12-12-S3.DOC]

Additional file 3: **The effects of time, space and their interaction on the clonal composition within taxa in Římov and Vír reservoirs.**

The table shows the results of multinomial generalised linear models, analysed in the R package, testing the effects of time, space and their interaction (as fixed factors) on clonal composition within taxa in each reservoir (i.e. on the response matrix containing the common multilocus genotypes and the “rare” category). The command “anova.multinom” was used to perform analyses of deviance.

| Taxon (reservoir) | Model | Residual DF | Residual Deviance | DF | LR statistic | *P* |
| --- | --- | --- | --- | --- | --- | --- |
| *D.galeata* (Římov) | time and space | 55 | 801.1 |  |  |  |
|  | time, space and their interaction | 50 | 787.2 | 5 | 13.94 | **0.016** |
|  | space | 60 | 922.7 |  |  |  |
|  | time and space | 55 | 801.1 | 5 | 121.64 | **< 0.001** |
|  | time | 60 | 980.9 |  |  |  |
|  | time and space | 55 | 801.1 | 5 | 179.78 | **< 0.001** |
| *D.galeata* (Vír) | time and space | 48 | 1110.9 |  |  |  |
|  | time, space and their interaction | 44 | 1089.8 | 4 | 21.02 | **< 0.001** |
|  | space | 52 | 1128.7 |  |  |  |
|  | time and space | 48 | 1110.9 | 4 | 17.86 | **0.001** |
|  | time | 52 | 1128.2 |  |  |  |
|  | time and space | 48 | 1110.9 | 4 | 17.34 | **0.002** |
| F1 hybrids (Vír) | time and space | 12 | 232.8 |  |  |  |
|  | time, space and their interaction | 10 | 221.9 | 2 | 10.89 | **0.004** |
|  | space | 14 | 247.2 |  |  |  |
|  | time and space | 12 | 232.8 | 2 | 14.46 | **< 0.001** |
|  | time | 14 | 242.4 |  |  |  |
|  | time and space | 12 | 232.8 | 2 | 9.69 | **0.008** |
| *D. longispina* (Vír) | time and space | 60 | 527.4 |  |  |  |
|  | time, space and their interaction | 55 | 512.5 | 5 | 14.92 | **0.011** |
|  | space | 65 | 612.9 |  |  |  |
|  | time and space | 60 | 527.4 | 5 | 85.53 | **< 0.001** |
|  | time | 65 | 599.0 |  |  |  |
|  | time and space | 60 | 527.4 | 5 | 71.65 | **< 0.001** |
